# Prevalence of intimate partner violence against women in Sweden and Spain: A psychometric study of the ‘Nordic paradox’

**DOI:** 10.1371/journal.pone.0217015

**Published:** 2019-05-16

**Authors:** Enrique Gracia, Manuel Martín-Fernández, Marisol Lila, Juan Merlo, Anna-Karin Ivert

**Affiliations:** 1 Department of Social Psychology, University of Valencia, Valencia, Spain; 2 Unit for Social Epidemiology, University of Lund, Malmö, Sweden; 3 Department of Criminology, Malmö University, Malmö, Sweden; Ben-Gurion University of the Negev Faculty of Health Sciences, ISRAEL

## Abstract

The high prevalence of intimate partner violence against women (IPVAW) in countries with high levels of gender equality has been defined as the “Nordic paradox”. In this study we compared physical and sexual IPVAW prevalence data in two countries exemplifying the Nordic paradox: Sweden (N = 1483) and Spain (N = 1447). Data was drawn from the European Union Agency for Fundamental Rights Survey on violence against women. To ascertain whether differences between these two countries reflect true differences in IPVAW prevalence, and to rule out the possibility of measurement bias, we conducted a set of analyses to ensure measurement equivalence, a precondition for appropriate and valid cross-cultural comparisons. Results showed that in both countries items were measuring two separate constructs, physical and sexual IPVAW, and that these factors had high internal consistency and adequate validity. Measurement equivalence analyses (i.e., differential item functioning, and multigroup confirmatory factor analysis) supported the comparability of data across countries. Latent means comparisons between the Spanish and the Swedish samples showed that scores on both the physical and sexual IPVAW factors were significantly higher in Sweden than in Spain. The effect sizes of these differences were large: 89.1% of the Swedish sample had higher values in the physical IPVAW factor than the Spanish average, and this percentage was 99.4% for the sexual IPVAW factor as compared to the Spanish average. In terms of probability of superiority, there was an 80.7% and 96.1% probability that a Swedish woman would score higher than a Spanish woman in the physical and the sexual IPVAW factors, respectively. Our results showed that the higher prevalence of physical and sexual IPVAW in Sweden than in Spain reflects actual differences and are not the result of measurement bias, supporting the idea of the Nordic paradox.

## Introduction

Intimate partner violence against women (IPVAW) remains a pervasive social and public health problem in western societies [1–8]. Increasing gender equality is at the core of the prevention efforts of this type of violence, as gender inequality is considered a main factor explaining IPVAW. Accordingly, rates of IPVAW are expected to drop as country-level gender equality increases [9–12] (see [13] for a review). However, in western countries, high country levels of gender equality are not always linked with low prevalence of IPVAW.

The high prevalence of IPVAW in countries with high levels of gender equality was defined by Gracia and Merlo as the “Nordic paradox” [14]. Nordic countries are, according to different international indicators (e.g., Global Inequality Index; Global Gender Gap Index; European Index of Gender Equality), the most gender equal countries in the world [15–17]. However, despite these high levels of gender equality, Nordic countries have high prevalence rates of IPVAW. The high prevalence of IPVAW in Nordic countries is illustrated by a European Union (EU) survey on violence against women conducted by the European Union Agency for Fundamental Rights (FRA) [18]. In this survey the average lifetime prevalence of physical and/or sexual violence by intimate partners in the 28 EU member states was 23%, with a range between 13% and 32%. However, Nordic countries in the EU were among the countries with higher lifetime prevalence of IPVAW, with rates of 32% (Denmark, the highest IPV prevalence in the EU), 30% (Finland), and 28% (Sweden). The high prevalence of IPVAW in Nordic countries is also supported by other studies and national surveys [19–25]. However, despite survey and research data pointing to a disproportionally high level of IPVAW in countries with the highest levels of gender equality like the Nordic ones, interestingly, this puzzling research question is rarely asked and, so far, remains unanswered.

The reasons explaining these high levels of IPVAW prevalence in Nordic countries, despite their high levels of gender equality, are not yet understood as almost no research has addressed specifically this paradox [22]. Gracia and Merlo [14], proposed a number of theoretical and methodological lines of inquiry towards understanding the Nordic paradox. However, as these authors noted [14], a first step to ascertain whether the Nordic paradox reflects true differences in IPVAW prevalence is to rule out the possibility that measurement bias is causing prevalence differences between Nordic and other countries. To eliminate this possibility, a key question is to ensure the comparability of IPVAW prevalence data across countries. In other words, comparisons of IPVAW data across countries should not be made without first ensuring measurement invariance.

IPVAW can be a culturally sensitive issue, and the way this type of violence is perceived or reported may vary across countries. Therefore, ensuring cross-cultural measurement invariance is critically important for appropriate and valid cross-cultural comparisons of self-reported IPVAW scores between respondents from different countries [26–32]. As Jang et al. noted [29], different perceptions of items or different interpretations of response scales can lead to measurement non-invariance (i.e., non-equivalence of measures). If this is the case, it cannot be assumed that the construct of interest, in our case IPVAW, is interpreted in the same way across countries because the same score in one country may have a different meaning or reflect different levels of IPVAW in another. Without ensuring measurement invariance, score comparisons across samples from different countries can be unreliable and inadequate, and the validity of comparing women’s IPVAW experiences across countries becomes questionable [28,29,32,33].

### Present study

Sweden and Spain are two countries exemplifying the Nordic paradox. According to several international gender equality indices, Sweden is ranked third in the Global Inequality Index [17], fifth in the Global Gender Gap Index [16], and first in the EU in the European Index of Gender Equality [15]. According to the same sources, Spain is ranked 13th (Global Inequality Index) or 24th (Global Gender Gap Index) in the world, and 11th in the EU (European Index of Gender Equality). However, despite the higher gender equality in Sweden, Spain has a substantially lower prevalence of IPVAW.

The FRA survey provides a composite indicator of the prevalence of physical and/or sexual violence by any partners (current and/or previous) since the age of 15. According to this indicator, the lifetime prevalence of physical and/or sexual violence among women perpetrated by any partner is 28% in Sweden and 13% in Spain ([18], p. 28). That is, the lifetime prevalence of physical and/or sexual IPVAW is 15 percentage points higher in Sweden than in Spain, while, according to the European Index of Gender Equality, gender equality in Sweden is 14 points higher than in Spain (the updated Gender Equality Index data for the year when the survey was conducted was 64 in Spain and 78 in Sweden, and is currently 68 in Spain and 82 in Sweden)[15].

One of the advantages of the FRA survey is that respondents from the 28 EU member states answer the same set of questions addressing different types of IPVAW. Another advantage of this survey is that it includes questions regarding IPVAW that are acts-based or behavioral oriented (e.g., being stabbed, cut, slapped, or being forced into sexual intercourse). This type of questions addressing IPVAW have a clear advantage over simply asking women whether their partners or ex-partners have ever been violent towards them, which is a more subjective approach and can lead to underreporting [4,34,35]. However, as the psychometric properties of the set of questions addressing physical and sexual IPVAW used in the FRA survey are unknown, and the measurement equivalence across countries (i.e., cross-cultural invariance) of these questions has never been tested, it is not possible to ascertain whether the differences between Sweden and Spain in lifetime prevalence of physical and sexual IPVAW reflect real differences or are the result of measurement bias.

A precondition to compare prevalence data on physical and sexual IPVAW across countries, in our case Sweden and Spain, is the availability of an equivalent measurement model. In this study, we aim to analyze whether the set of questions assessing physical and sexual IPVAW used in the FRA survey are reliable, valid and comparable measures of these types of violence across Sweden and Spain. If the measures of physical and sexual IPVAW are comparable and confirm higher levels of physical and sexual violence in Sweden than in Spain, these results would support the idea that the Nordic paradox (at least with respect to Sweden and Spain) reflects real prevalence differences.

## Method

### Participants

In this study we used data from the European Union Agency for Fundamental Rights on violence against women. This data is deposited in the UK Data Service, and the study has been approved by European Union Agency for Fundamental Rights, who granted a Special License for secondary data analysis (Reference No. 102577) to Enrique Gracia as Principal investigator of the project and first and corresponding author of this paper.

For this study, we used the Spanish (N = 1447) and Swedish (N = 1483) samples from the survey conducted by the European Union Agency for Fundamental Rights on violence against women [18]. Respondents to this survey were ever-partnered women, aged 18 to 74. The sampling followed a two-stage clustered stratified design with the same probability of selection of households within clusters. The responses were collected in-person in both countries, although in Sweden the first contact was made telephonically. Further details on sample collection and procedures can be found in FRA [36]. Socio-demographical variables of both samples are described on Table 1.

**Table 1:** Socio-demographical variables (unweighted).

	Total (%)	Spain (%)	Sweden (%)
Age			
18–24	127 (4.3)	77 (5.3)	50 (3.4)
25–29	164 (5.6)	102 (7.0)	62 (4.2)
30–34	218 (7.4)	122 (8.4)	96 (6.5)
35–39	270 (9.2)	163 (11.2)	107 (7.2)
40–49	662 (22.6)	335 (23.2)	327 (22.0)
50–59	624 (21.3)	272 (18.8)	352 (23.7)
60+	865 (29.5)	376 (26.0)	489 (33.0)
Income			
Finding it very difficult on present income	172 (5.9)	132 (9.1)	40 (2.7)
Finding it difficult on present income	436 (14.9)	263 (18.2)	173 (11.7)
Coping on present income	1226 (41.8)	608 (42.0)	618 (41.7)
Living comfortably on present income	1075 (36.7)	428 (29.6)	647 (43.6)
Education			
Has not completed primary education	250 (8.5)	75 (5.2)	4 (0.3)
Completed primary education	719 (24.5)	221 (15.3)	62 (4.2)
Compulsory secondary education	422 (14.4)	461 (15.3)	84 (5.7)
Upper secondary education	620 (21.2)	282 (31.9)	331 (22.3)
Post-secondary education (but not university level)	549 (18.7)	135 (19.5)	280 (18.9)
Graduate studies	286 (9.8)	219 (15.1)	493 (33.2)
Post-graduate studies	250 (8.5)	53 (3.7)	195 (13.1)
Self-perceived Health			
Very Bad	28 (1.0)	16 (1.1)	12 (0.8)
Bad	173 (5.9)	94 (6.5)	79 (5.3)
Fair	555 (18.9)	295 (20.4)	260 (17.5)
Good	1254 (42.8)	684 (47.3)	570 (38.4)
Very Good	916 (31.3)	354 (24.5)	562 (37.9)
My partner or an ex-partner has been physically violent against me			
Yes	404 (15.9)	137 (9.7)	267 (23.6)
No	2143 (84.1)	1280 (90.3)	863 (76.4)
My partner or an ex-partner has been sexually violent against me			
Yes	127 (5.0)	59 (4.2)	68 (6.0)
No	2416 (95.0)	1357 (95.8)	1059 (94.0)

### Measures

#### Physical violence

The FRA survey included 10 items addressing physical IPV perpetrated by the current or any previous partner (e.g., “Your current/previous partner has slapped you?”, “Your current/previous partner has grabbed you or pulled your hair?”). Participants have to answer in a 4-point Likert-type scale indicating how often have they experienced this type of violence (1: “Never”, 2: “Once”, 3: “2–5 times”, 4: “6 or more times”). In this study respondents were considered victims of intimate partner physical violence when reported one of the episodes described by the items at least one time, whereas severe violence was considered in those cases where respondents have experienced the episodes more than one time.

#### Sexual violence

FRA survey addresses intimate partner sexual violence with 4 items describing episodes of sexual violence perpetrated by the current or any previous partner (e.g., “Your current/previous partner has forced you into sexual intercourse by holding you down or hurting you in some way?”, “Your current/previous partner has made you take part in any form of sexual activity when you did not want to or you were unable to refuse?”). Respondents have to indicate how often have they experienced this type of violence using a 4-point Likert-type (1: “Never”, 2: “Once”, 3: “2–5 times”, 4: “6 or more times”). Respondents were considered as victims of intimate partner sexual violence when reported one of the episodes described by the items at least one time, whereas severe violence was considered in those cases where respondents have experienced the episodes more than one time.

#### Validity evidence based on relations to other variables

To test validity based on relations to other variables [37], we used two measures: (1) *Self-perceived health*. The FRA survey included an item in which respondents are asked how their health was in general, and they have to answer using a 5-point Likert-type ordinal scale (ranging from 1 = “Very Bad” to 5 = “Very Good”). “Do not know”, “Not applicable” and “Refused” categories were treated as missing values. (2) *Self-reported physical and sexual IPVAW victimization*. At the end of the FRA survey, respondents are asked to complete, confidentially, two dichotomous items (Yes/No) about experienced life-time physical IPV (“My partner or an ex-partner has been physically violent against me”), and experienced life-time sexual IPV (“My partner or an ex-partner has been sexually violent against me”).

### Data analyses

First, descriptive analyses of the set of items assessing physical and sexual violence included in the FRA survey were conducted. The mean, standard deviation, skewness, and kurtosis statistics were computed for each item. These statistics were obtained with the unadjusted responses of the participants, as the aim was to study the properties of the items.

A confirmatory factor analysis (CFA) was conducted to assess the latent structure (i.e., internal construct validity) of the set of questions used in the FRA survey to address physical and sexual violence. Two models were estimated and compared using robust weighted least squares (WLSMV), as this method tend to perform better with categorical data [38]. The first model was a one-factor model in which all items loaded onto a single violence factor, implying that all violent acts, regardless of their physical or sexual nature, pertained to the same construct. The second model was a two-factor model where the items addressing physical violence loaded on one factor and the items assessing sexual violence on another factor, implying that each set of items were sampling different constructs. In this second model the factors are correlated, and thus these two constructs are assumed to be related. Model fit was tested with a combination of fit indices: comparative fit index (CFI), Tucker-Lewis index (TLI), and root mean square error of approximation (RMSEA). CFI and TLI values above .95 are indicative of good fit, whereas RMSEA values below .08 and .06 are considered indicative of mediocre and good fit, respectively [39,40]. Once the latent structure is determined, the internal consistency of the resulting factor or factors will be studied by computing Cronbach’s α and McDonald’s ω. McDonald’s ω is more suitable when the items are not tau-equivalent (i.e., they do not have the same factor loadings) [41]. After establishing the latent structure of the items, validity based on relationships with other variables was tested conducting a set of mean comparisons and correlations with variables with expected links to IPVAW (i.e., self-perceived health, and self-reported physical and sexual IPVAW victimization).

Once these analyses have been carried out separately for both Sweden and Spain, to ensure the comparability of IPVAW scores across these countries two complementary analyses were conducted: A differential item functioning (DIF) analysis for categorical data, and a multi-group confirmatory factor analysis (MG-CFA) between countries to test measurement invariance [42–45]. Both procedures aim to assess whether there is a group effect (i.e., country) on IPVAW factor scores, but they focus on different issues. Whereas the DIF focuses on the equivalence of the latent scores, the MG-CFA focuses on the equivalence of the structural parameters of the model (e.g., loadings and intercepts). First, a DIF analysis was conducted using the logistic regression method [46,47]. An item presents DIF when the probability of endorsement of an item category is not the same for respondents from different groups (i.e., countries) with equivalent scores in the factor, indicating that the respondents of each group are answering that item differentially. Second, a series of MG-CFA was conducted, testing configural, metric, and scalar measurement invariance levels across the Swedish and Spanish samples [27,48–51]. These levels of invariance are required for a meaningful comparison of IPVAW scores for Sweden and Spain. Configural invariance evaluate whether Swedish and Spanish women conceptualize the construct in the same way, testing if the same factorial model fits for both groups. Metric invariance constraint the factor loadings to be equal across groups, implying that Swedish and Spanish respondents interpret the items similarly. Scalar invariance test whether the same threshold parameters could be estimated for each group, indicating that the items yield the same factor score for Swedish and Spanish samples. Change in CFI (ΔCFI) and RMSEA (ΔRMSEA) was computed to test which of these invariance levels were better supported by the data. If the change in the CFI (ΔCFI) and in the RMSEA (ΔRMSEA) is below .010 or .015, respectively, then the most restrictive level of invariance is supported [26,52].

After assessing measurement invariance, the raw prevalences of the items were compared as a descriptive analysis of the differences between Sweden and Spain. Finally, a MG-CFA latent means analysis was also conducted, to analyze IPVAW differences across countries. Factor scores on latent variables provides a more refined approach to assess differences in IPVAW between two countries. They are continuous variables that take into account how relevant for the factor is each item, and can capture more variability. To assess the magnitude of the latent mean differences, Cohen’s *d* effect size index was obtained using the resulting factor scores [49]. Cohen *d* can also be used to compute the Cohen *U*_*3*_ statistic, which evaluate the percentage of cases of one group that is higher than the average of the other group, and the probability of superiority, which indicates the probability that a person selected at random from one group will have a higher score than a person randomly selected from the other group [53–55].

Descriptive, DIF, and validity analyses were carried out with the statistical software package R [56], using the *psych* and *lordif* libraries [46,57]. The CFA and the measurement invariance analyses were conducted with the software package *Mplus* [58].

## Results

### Descriptive analyses

The descriptive statistics of the items addressing physical violence can be found in Table 2. The means of the items were around 1, the lowest category (i.e., “Never”), with standard deviations around 0.4 and 0.5 for the Spanish and Swedish women, respectively. Both groups presented positive skew statistics and high kurtosis values, indicating that most of the responses were centered in the lower categories. The variance of the items 7 and 9 (i.e., “being burned”, and “being cut, stab or shot”) was extremely low, indicating that almost none of the respondents reported experiencing this type of violence. Given the lack of variability in the responses on these items in both countries (1% or less), they were removed for subsequent analyses.

**Table 2:** Physical violence items descriptive statistics.

Spain				
	*M*	*SD*	*Skew*	*Kurtosis*
Threatened to hurt you physically?	1.19	0.66	3.55(.02)	11.39(.02)
Pushed you or shoved you?	1.20	0.65	3.39(.02)	10.46(.02)
Slapped you?	1.13	0.55	4.28(.01)	17.61(.01)
Thrown a hard object at you?	1.07	0.40	6.25(.01)	39.70(.01)
Grabbed you or pulled your hair?	1.09	0.45	5.24(.01)	27.54(.01)
Beat you with a fist or a hard object, or kicked you?	1.08	0.44	5.55(.01)	30.97(.01)
Burned you?	1.01	0.11	18.91(.00)	356.01(.00)
Tried to suffocate you or strangle you?	1.04	0.26	8.26(.01)	75.82(.01)
Cut or stabbed you, or shot at you?	1.01	0.11	18.73(.00)	407.65(.00)
Beat your head against something?	1.04	0.29	8.15(.01)	70.02(.01)
Sweden				
	*M*	*SD*	*Skew*	*Kurtosis*
Threatened to hurt you physically?	1.30	0.79	2.53(.02)	5.08(.02)
Pushed you or shoved you?	1.37	0.83	2.12(.02)	3.19(.02)
Slapped you?	1.21	0.65	3.2(.02)	9.36(.02)
Thrown a hard object at you?	1.13	0.52	4.24(.01)	17.79(.01)
Grabbed you or pulled your hair?	1.17	0.61	3.64(.02)	12.31(.02)
Beat you with a fist or a hard object, or kicked you?	1.16	0.60	3.84(.02)	13.80(.02)
Burned you?	1.01	0.10	22.90(.00)	616.22(.00)
Tried to suffocate you or strangle you?	1.05	0.29	7.23(.01)	56.92(.01)
Cut or stabbed you, or shot at you?	1.00	0.07	15.24(.00)	230.51(.00)
Beat your head against something?	1.08	0.40	5.47(.01)	31.10(.01)

Note: M = Mean, SD = Standard Deviation, In brackets: Skew and Kurtosis statistics standard error.

Regarding the sexual violence items (Table 3), the means were also centered on the lower category (i.e., “Never”), with standard deviations around 0.40 and 0.50 for the Spanish and the Swedish respondents, and showed a positive skew and had high kurtosis values. As in the physical violence items, the respondents tended to select the lower categories in the sexual violence items.

**Table 3:** Sexual violence items descriptive statistics.

SPAIN				
	*M*	*SD*	*Skew*	*Kurtosis*
has forced you into sexual intercourse by holding you down or hurting you in some way?	1.06	0.38	6.86(.01)	47.00(.01)
has attempted to force you into sexual intercourse by holding you down or hurting you in some way?	1.05	0.36	7.31(.01)	53.59(.01)
has made you take part in any form of sexual activity when you did not want to or you were unable to refuse?	1.05	0.35	7.57(.01)	57.29(.01)
Have you consented to sexual activity because you were afraid of what your current partner might do if you refused?	1.06	0.39	6.85(.01)	46.56(.01)
SWEDEN				
	*M*	*SD*	*Skew*	*Kurtosis*
has forced you into sexual intercourse by holding you down or hurting you in some way?	1.10	0.48	4.91(.01)	23.65(.01)
has attempted to force you into sexual intercourse by holding you down or hurting you in some way?	1.09	0.45	5.15(.01)	26.49(.01)
has made you take part in any form of sexual activity when you did not want to or you were unable to refuse?	1.13	0.54	4.17(.01)	16.76(.01)
Have you consented to sexual activity because you were afraid of what your current partner might do if you refused?	1.11	0.51	4.88(.01)	22.95(.01)

Note: M = Mean, SD = Standard Deviation. In brackets: Skew and Kurtosis statistics standard error.

### Confirmatory factor analysis and internal consistency

A one-factor model and a two-factor model were then estimated to determine the latent structure of the items for each country separately, using WLSMV as the estimation method. Both models converged successfully.

**Fig 1:**
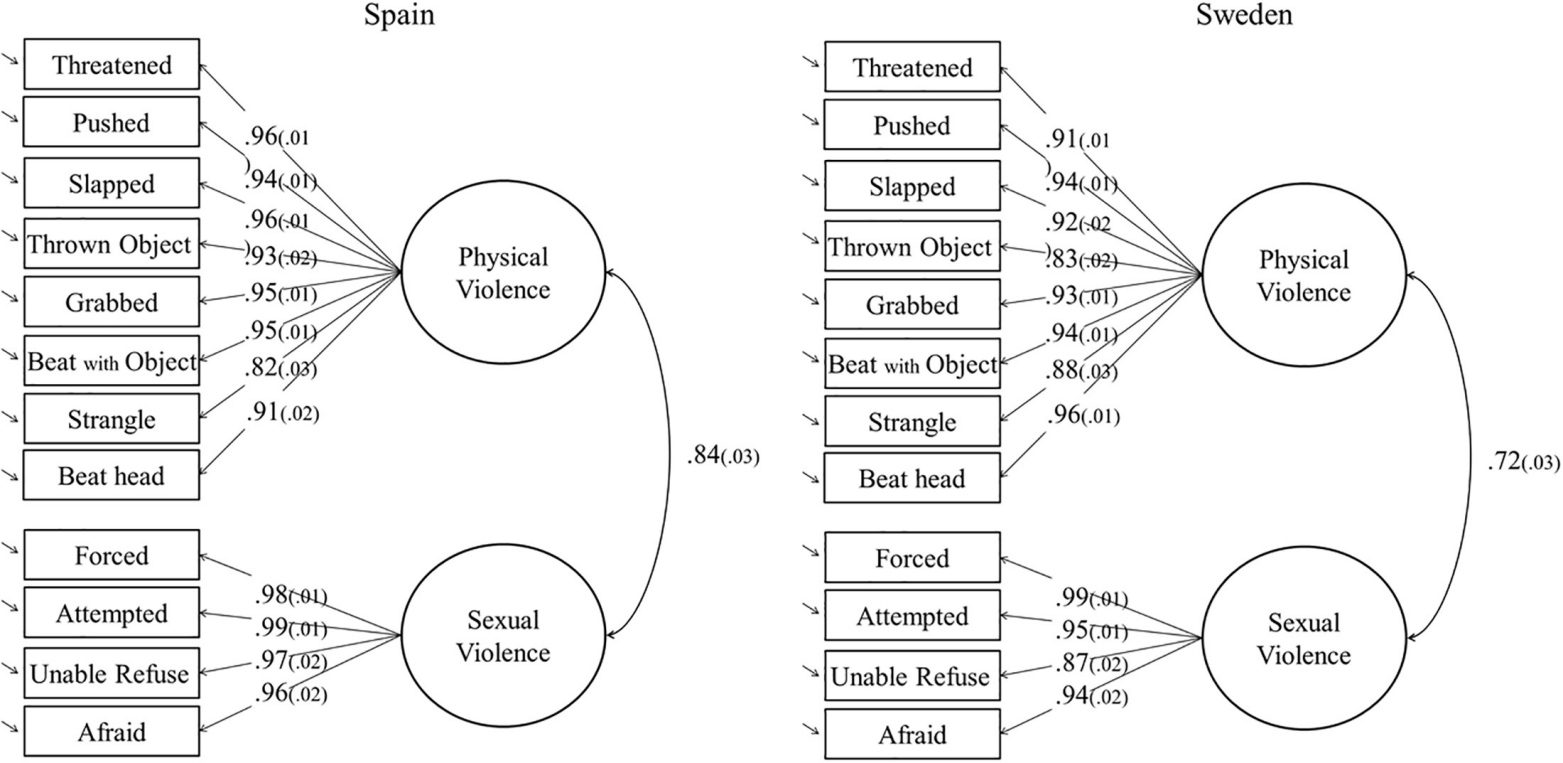
CFA two-factor model.

The one- and two-factor models fitted adequately in the Spanish sample (Table 4). In the Swedish sample the one-factor model showed a good fit to the data, although the RMSEA was mediocre. Adding a second factor improved substantially the RMSEA in the Swedish group, being below the .06 cut-off for a well fitted model. For this reason, we decided to keep the two-factor solution in both samples, as both countries showed similar fit indices. The factor loadings of the items in the Spanish and the Swedish samples were high, showing values above .80 in both factors. This indicates that the items were strongly related to the measured construct. The correlations between the factors were also high, .84 and .72 for the Spanish and the Swedish groups, respectively (Fig 1).

**Table 4:** CFA fit indices.

Model		χ^2^	*df*	CFI	TLI	RMSEA [95% CI]
One-factor	Spain	243.24	54	0.99	0.99	0.049 [0.043; 0.056]
	Sweden	630.74	54	0.97	0.97	0.086 [0.080; 0.092]
Two-factor	Spain	125.51	53	0.99	0.99	0.031 [0.024; 0.038]
	Sweden	159.71	53	0.99	0.99	0.037 [0.030; 0.037]

Note: CFI: Comparative fit index, TLI: Tucker-Lewis index, RMSEA = Root mean squared error of approximation.

Regarding the internal consistency, both factors showed a high internal consistency. In particular, the physical IPVAW factor showed a Cronbach’s α = .91 in both countries, and a McDonald’s ω = .92 in the Spanish sample and .91 in the Swedish sample. The sexual IPVAW factor had a Cronbach’s α = .88 and .86, and a McDonald’s ω = .90 and .86 in the Spanish and Swedish groups, respectively.

### Validity evidence based on relations to other variables

The standardized factor scores from the two-factor model were used to conduct the validity analyses, as the items did not contribute equally to their factor (i.e., are not tau-equivalent).

The scores on the physical IPVAW factor were compared by self-perceived health categories in each country separately. In Spain, we found significant differences in this factor, *F*(4) = 6.39, *p* < .001, *η2* = .017. Post-hoc analysis showed that the differences in self-perceived health were between the upper two categories (i.e., “Very good” and “Good”) and the lower two categories (i.e., “Bad” and “Very bad”), implying that respondents who indicated a positive self-perceived health showed lower scores on this factor. We also found significant differences in the Swedish sample by self-perceived health, *F*(4) = 10.26, *p* < .001, *η*^*2*^ = .027. We found in the post-hoc analyses that respondents who chose the upper category in self-perceived health (i.e. “Very Good”) showed lower scores in the physical IPVAW factor in comparison with the other response categories.

Regarding the sexual IPVAW factor, we found significant differences in the Spanish sample when the scores in this factor were compared by self-perceived health categories, *F*(4) = 6.63, *p* < .001, *η*^*2*^ = .018. In particular, post-hoc analyses indicated that respondents who chose the lowest category of self-perceived health (i.e. “Very Bad”) showed higher scores on the sexual IPVAW factor than those respondents who chose the upper three categories (i.e., “Very Good”, “Good”, and “Fine”). Significant differences by self-perceived health were also found in the Swedish sample, *F*(4) = 6.52, *p* < .001, *η*^*2*^ = .017. These differences were between the upper two and the lower two categories of this variable (i.e., “Very Good” and “Good” vs. “Very Bad” and “Bad”).

The scores on both physical and sexual violence IPVAW factors were also correlated with the self-reported physical and sexual IPVAW victimization items of the FRA survey for each country separately. Biserial correlations were used. We found that the physical violence factor scores were positively related to the single item of the FRA survey addressing physical violence in Sweden and Spain (*r* = .33, *p* < .001, and *r* = .37, *p* < .001, respectively). The scores on this factor were also related to the sexual violence item, especially in Spain (*r* = .16, *p* < .001, in Sweden, and *r* = .35, *p* < .001, in Spain). Regarding the sexual violence, we found a positive relationship between the factor scores and the single item of the FRA survey in both countries (*r* = .38, *p* < .001, in Sweden, and *r* = .26, *p* < .001, in Spain). The correlations between the sexual violence factor scores and the single item addressing physical violence from the FRA survey were also positive in Sweden and Spain (*r* = .26, *p* < .001, and *r* = .25, *p* < .001, respectively).

### Measurement equivalence analyses

First, a series of nested logistic regression models for categorical data was conducted to identify, at item-level, if there was a group effect due to pertaining to different countries for both factors, and whether that effect was constant—uniform DIF—or varies across the factor scores—non-uniform DIF—[46]. We detected non-uniform DIF only for item 4 (i.e., “Thrown a hard object at you”) in the physical IPVAW factor, χ^2^(1) = 12.11, *p* < .001, *R*^*2*^_*Nagelkerke*_ = .008. The effect size in this item was, however, below 0.02, and thus this could be considered negligible, as adding the DIFF effect to the model does not improve their fit substantially [46,53]. No DIF was detected for any items of the sexual IPVAW factor.

Second, measurement invariance across countries was explored using a MG-CFA for the two-factor model. The configural, metric, and scalar invariance levels were tested (see Table 5). The configural invariance level was supported by the data, entailing that the same factorial model can be applied in both countries. Constraining the factor loadings to have the same value in both groups did not substantially decrease the fit of the CFI and the RMSEA fit indices (ΔCFI = .000, ΔRMSEA = .001), indicating that the metric invariance level could be assumed. Finally, when the threshold parameters of the items were constrained to be equal across groups, the change of the CFI and the RMSEA indices were below the ΔCFI = .10 and ΔRMSEA = .15 cut-offs, supporting the scalar invariance level.

**Table 5:** Measurement Invariance fit indices.

	χ^2^	*df*	CFI	TLI	RMSEA [95% CI]	
*Invariance level*						
Configural	281.80	106	0.996	0.996	0.034	[0.029; 0.038]
Metric	305.73	116	0.996	0.996	0.033	[0.029; 0.038]
Scalar	551.18	152	0.992	0.993	0.042	[0.039; 0.046]
Latent Means	352.31	150	0.996	0.996	0.030	[0.026; 0.034]

Note: CFI: Comparative fit index, TLI: Tucker-Lewis index, RMSEA = Root mean squared error of approximation.

### Raw prevalences

As no DIF was detected, item-based comparisons across countries can be made. All items, both in the physical and sexual IPVAW factors, had a higher prevalence in Sweden than in Spain (Table 6). These differences held for both general prevalence (physical IPVAW: Sweden: 7.9%, Spain: 4.3%; sexual IPVAW: Sweden: 5.5%, Spain: 2.3%) and severe prevalence (physical IPVAW: Sweden: 5.1%, Spain: 2.8%; sexual IPVAW: Sweden: 4%, Spain: 1.8%). As for the raw prevalence considering all items, in Sweden the prevalence of physical and sexual IPVAW was also higher than in Spain.

**Table 6:** Physical and sexual violence item prevalences.

	Spain		Sweden	
	Prev (%)	Sev Prev (%)	Prev (%)	Sev Prev (%)
*Physical Violence*				
Threatened to hurt you physically?	8.43	6.35	15.30	10.85
Pushed you or shoved you?	9.95	6.50	19.84	13.16
Slapped you?	6.56	4.49	11.59	6.94
Thrown a hard object at you?	3.52	2.14	7.35	4.25
Grabbed you or pulled your hair?	4.77	3.04	8.62	6.20
Beat you with a fist or a hard object, or kicked you?	4.22	2.76	7.88	5.59
Tried to suffocate you or strangle you?	2.49	0.09	3.10	1.35
Beat your head against something?	2.21	1.24	4.78	2.62
Total	12.43	8.03	27.86	16.76
*Sexual Violence*				
has forced you into sexual intercourse by holding you down or hurting you in some way?	2.63	1.94	5.25	3.70
has attempted to force you into sexual intercourse by holding you down or hurting you in some way?	2.21	1.80	4.85	3.43
has made you take part in any form of sexual activity when you did not want to or you were unable to refuse?	2.00	1.73	7.14	4.98
have you consented to sexual activity because you were afraid of what your current partner might do if you refused?	2.49	2.00	4.98	4.04
Total	4.28	3.09	10.90	7.45

### Latent means analysis

Once the scalar invariance level was established, the differences between Spanish and Swedish women were assessed estimating a new MG-CFA. This model assumes that the structural parameters (i.e., slopes and thresholds) are equal, and thus the means of the factor scores can be compared assuming that respondents interpret the items similarly in both groups. For the Spanish sample the mean was fixed to zero in both physical and sexual IPVAW factors, whereas in the Swedish sample these parameters were freed. The Swedish sample showed a higher latent mean in the physical IPVAW factor than the Spanish sample (*z* = 0.72, *p* < .001). The effect size of these differences between Sweden and Spain was large, *d* = 1.23, *Cohen’s U*_*3*_ = .891, *probability of superiority* = .807. This means that 89.1% of the Swedish sample presented higher values in the physical IPVAW factor than the average of the Spanish sample, and if one woman is randomly selected from each country, there is an 80.7% probability that the Swedish woman will score higher in this factor than a Spanish woman.

Regarding the sexual violence factor, we found that the latent mean was also higher in the Swedish group (*z* = 1.99, *p* < .001). In this case the effect size was extremely large, *d* = 2.5, *Cohen’s U*_*3*_ = .994, *probability of superiority* = .961, which means that the 99.4% of the Swedish women presented higher values on the sexual IPVAW factor scores than the Spanish women. Also, if one woman is randomly selected from each country, there is a 96.1% probability that the Swedish woman will score higher in the sexual IPVAW factor than a Spanish woman.

## Discussion

In this study we compared physical and sexual IPVAW prevalence data in two countries exemplifying the Nordic paradox [14]: Sweden and Spain. To ascertain whether differences between these two countries reflect true differences in IPVAW prevalence, and to rule out the possibility of measurement bias, we conducted a set of analyses to ensure measurement equivalence, as a precondition for appropriate and valid cross-cultural comparisons. Once an equivalent measurement model had been established, we compared physical and sexual IPVAW scores between the two countries. Our results showed that the higher levels of physical and sexual IPVAW in Sweden than Spain reflect actual differences in IPVAW prevalence and are not the result of measurement bias, supporting the idea of the Nordic paradox.

The first set of analyses conducted in this study aimed to examine whether the series of questions assessing physical and sexual IPVAW used in the FRA survey were reliable and valid measures of this type of violence in both Sweden and Spain. First, results from CFA examining the latent structure of the items used in the FRA survey supported a two-factor model in the two countries. That is, these items were measuring two separate constructs: physical and sexual IPVAW. Once the latent structure of the physical and sexual violence items had been established, reliability analyses (computing Cronbach’s α and McDonald’s ω) were conducted, showing that these scales had high internal consistency in both countries (all values ranging from .86 to .92). In this first set of analyses, we also addressed the validity of physical and sexual IPVAW factors based on their relations to other variables in the two countries. In both Sweden and Spain, scores in the physical and sexual IPVAW factors were significantly associated, as expected, to self-perceived health. The physical and sexual IPVAW scores were also correlated with two single-item measures of self-reported (not act-based measures) physical and sexual IPVAW victimization.

Once the psychometric properties of these measures had been established for each country, the next set of analyses aimed to ensure the comparability of these measures across Sweden and Spain by conducting different measurement equivalence tests. In the present study, to test the comparability of the physical and sexual IPVAW scales between Sweden and Spain, two complementary analyses were conducted: a DIF analysis and a MG-CFA. The joint use of these two techniques is one of the main strengths of the current manuscript, as they provide complementary information. In particular, both analyses showed that the country had no effect on the physical and the sexual IPVAW scores. No DIF was detected, indicating that the probability of endorsing a category of response in each item was the same for Swedish and Spanish respondents and, therefore, factors scores were comparable (i.e., no recalibration of item parameters was needed). Regarding MG-CFA, configural, metric, and scalar measurement invariance levels were supported, indicating that respondents in Sweden and Spain used the same conceptual framework to respond to the items (i.e., configural invariance), that the items were interpreted in a similar way, contributing equally to the scale scores (i.e., metric invariance), and that differences across countries in the observed items were the result of actual differences in the corresponding latent factors of physical and sexual IPVAW (i.e., scalar invariance). Results from these measurement invariance analyses ensured the comparability of physical and sexual IPVAW scores between Spanish and Swedish respondents.

When we examined the raw prevalence of the items, both in the physical and sexual IPVAW scales, all had a higher prevalence in Sweden than in Spain (both for general and severe IPVAW). Considering all items together, the general lifetime prevalence of IPVAW was higher in Sweden (physical: 27.86%, sexual: 10.9%) than in Spain (physical: 12.43%, sexual: 4.3%). The same pattern was also found for severe physical (16.76% Sweden vs. 8.03% Spain) and sexual (7.4% Sweden vs. 3.1% Spain) IPVAW. However, although comparisons based on the raw prevalence can be useful as a first descriptive step, they provide a limited description of the phenomenon, as this measure does not consider the differential contribution of each item to its corresponding factor (i.e., not all items have the same importance), and cannot capture as much variability as a continuous measure like the factor scores on latent variables.

Latent means comparisons between the Spanish and the Swedish samples showed that the standardized factor scores on both the physical and sexual IPVAW factors were higher in Sweden than in Spain, and that these differences were substantially higher for sexual IPVAW. The effects size of these differences was large for both types of IPVAW, and particularly remarkable in the case of sexual IPVAW. If we transform the effect size into percentages, 89.1% of the Swedish sample had higher values in the physical IPVAW factor than the Spanish average, whereas 99.4% of the Swedish women presented higher values in the sexual IPVAW than the Spanish latent mean in that factor. When we analyze these effect sizes in terms of probability of superiority (i.e., the probability that a woman from one country will score higher than a woman from the other country, if both are randomly selected), there was an 80.7% probability that a Swedish woman would score higher than a Spanish woman in the physical IPVAW factor, and a 96.1% probability that the Swedish woman would score higher than the Spanish woman in the sexual IPVAW factor. These results clearly illustrate the importance of using appropriate measurement approaches for cross-country comparisons, as they provide a more accurate picture of country differences. Prevalence indicators based on raw prevalences provide a more restricted view of the phenomenon, and can distort or conceal important differences, such as those found in this study regarding sexual IPVAW differences between Sweden and Spain.

Summing up, our results showed that the prevalence of physical and sexual IPVAW is clearly higher in Sweden than in Spain, that these differences are more evident in the case of sexual violence, and that these differences are not the result of measurement bias. Taken together, these results support the idea of the Nordic paradox, that is, the puzzling fact that despite the high levels of gender equality achieved in countries like Sweden, the prevalence of physical and, in particular, sexual IPVAW remains disproportionately high. The higher rates of physical and sexual IPVAW in countries with high levels of country-level gender equality such as Sweden––regardless of whether we consider its prevalence on its own, or in comparison with another country with lower levels of gender equality such as Spain––remains unexplained, and clearly invites further research. The psychometric study conducted in this paper was not designed to explain the Nordic paradox, but to eliminate the possibility that this phenomenon was due to measurement bias. Once measurement bias has been ruled out, the research question posited by the Nordic paradox remains unanswered.

The reasons explaining the high levels of IPVAW prevalence in countries like Sweden, despite their high levels of gender equality, are not yet understood. Although research supports the link between country-level gender equality and violence against women, the nature and direction of this relationship appears to be complex [13]. For example, a systematic review analyzed the evidence supporting different hypotheses regarding the relationships between country-level gender equality and violence against women: increased gender equality decreased violence (amelioration hypotheses), increased gender equality increased violence (backlash hypotheses), and increased gender equality equals men and women in experiencing violence (convergence hypothesis [59]. This review concluded that none of these relationships could be assumed, and that this association is complex and should be further investigated. For example, to shed light on the Nordic paradox, future research should examine a number of potential lines of enquiry such as those proposed by Gracia and Merlo [14]. Future research should also extend the type of analysis conducted in this study to include other Nordic countries, as well as other countries with low levels of gender equality and also lower levels of IPVAW. This type of research should acknowledge the complex and multidetermined nature of IPVAW [60–62], with appropriate methodological approaches such as multilevel analyses of individual heterogeneity and discriminatory accuracy [63–65].

This study has clear implications regarding cross-country comparisons on key issues such as IPVAW. For adequate cross-cultural comparisons, international surveys should use reliable and valid measures, and most importantly, ensure measurement invariance. Establishing cross-cultural measurement invariance is a precondition for appropriate and valid comparisons across countries [26,28–32]. As Davidov noted [48], “absent invariance, observed differences in means or other statistics might reflect differences in systematic biases of response across countries or different understanding of the concepts, rather than substantive differences” (p. 429). Lack of evidence of measurement invariance can cast doubts on how cross-country comparisons are interpreted. Using reliable, valid and comparable measures (i.e., using an equivalent measurement model) prevents uncertainty or ambiguous interpretations, and ensures that we reach the right conclusions when comparing countries on key issues such as IPVAW.
